# Effects of increasing levels of rubber seed cake on growth performance, nutrient digestion metabolism, serum biochemical parameters, and rumen microbiota of Hu sheep

**DOI:** 10.1186/s12917-025-04503-7

**Published:** 2025-02-05

**Authors:** Huwei Zhao, Jinling Hua, Wenwen Lu, Longfei Yan, Min Zhang, Chao Chen, Xiaokang Lv

**Affiliations:** College of Animal Science, Anhui Science and Technology University, Chuzhou, 239000 China

**Keywords:** Rubber seed cake, Growth performance, Nutrient digestion metabolism, Serum biochemical parameters, Rumen microbiota

## Abstract

This study aimed to reveal the effects of increasing levels of rubber seed cake (RSC) on growth performance, nutrient digestion metabolism, serum biochemical parameters, and rumen microbiota in Hu sheep. In this study, 48 Hu sheep, weighing 17.01 ± 0.57 kg at 3 months of age, were randomly divided into four treatments: 0% rubber seed cake (RSC0%), 6% rubber seed cake (RSC6%), 12% rubber seed cake (RSC12%) and 18% rubber seed cake (RSC18%), with 12 sheep per group. Compared to the RSC0%, the ADG and DMI of the RSC6% and RSC12% were increased (*P* > 0.05). The apparent digestibility of OM and EE quadratically (*P* < 0.05) changed with the increase of RSC supplementation, with the greatest apparent digestibility of OM and EE observed in the RSC6% diet. With increased RSC supplementation, the N intake and fecal N increased linearly (*P* < 0.05), and the apparent digestibility of N reduced linearly (*P* < 0.05). As the increase of RSC supplementation, the serum levels of IgA, IgM, IgG, IL-4, T-AOC, and GSH-Px increased linearly (*P* < 0.05), and the serum level of IL-6 reduced linearly (*P* < 0.05). The serum level of IL-1β reduced quadratically (*P* < 0.05) with the increased RSC dose, and the serum level of SOD increased quadratically (*P* < 0.05) with the increased RSC dose. The ruminal NH_3_-N and the relative abundance of *norank_Muribaculaceae* quadratically (*P* < 0.05) changed with increased RSC supplementation, and the greatest relative abundance of *norank_Muribaculaceae* was observed in the RSC6% diet. In general, incorporating RSC into the diet of Hu sheep did not adversely affect growth performance and rumen fermentation characteristics. Supplementing with 6% RSC enhanced the relative abundance of *norank_Muribaculacea* in the rumen fluid and the immune and antioxidant capabilities. However, supplementing with 12 and 18% RSC might have negatively impacted nutrient digestion and metabolism. Therefore, this study recommended replacing corn and soybean meal with 6% RSC in the diet of Hu sheep.

**Supplementary Information:**

The online version contains supplementary material available at 10.1186/s12917-025-04503-7.

## Introduction

Corn and soybean meal (SBM), recognized as high-quality sources of energy and protein, constitute a substantial component of livestock and poultry diets. The volatility in imported supplies and the persistent escalation of prices not only amplify feed costs but also introduce considerable risks to the sustainability of the livestock industry and the stability of food security [1, 2]. Therefore, exploring and applying innovative feed resources to reduce dependence on corn and SBM is of great significance to livestock husbandry and food security.

The rubber tree originated in Brazil and is now cultivated extensively in Southeast Asia [3, 4]. Xishuangbanna Prefecture in China is a key region for rubber plantations [5]. In 2023, global natural rubber production reached 14.32 million tons. Despite the high production of rubber seeds, their utilization rate is disappointingly low, with only 25% used for breeding, leaving 75% discarded, leading to a significant waste of resources. RSC is a by-product of oil extraction from rubber seed kernel, contains 20%-25% protein and 2,520 kcal/kg energy [6], and trace minerals like iron (180 mg/kg), copper (50 mg/kg), manganese (90 mg/kg), and zinc (150 mg/kg), with zinc content three times higher than that found in SBM [7]. Moreover, RSC contains a balanced and rich array of essential amino acids [8]. A recent study indicated that supplementing 15% RSC as a substitute for fish meal in the diet did not significantly affect the growth performance, digestive function, and antioxidant capacity of *Hemibagrus wyckioides* [9]. Furthermore, a diet containing 150 g/kg RSC did not negatively influence tilapia's weight gain and daily growth coefficient; however, increased RSC levels were correlated with reduced growth rate, digestive enzyme activity, and antioxidant capacity [10]. The appropriate supplementation of RSC to the feed did not impact the feeding rate of Nile tilapia at 50% [11] and Labeo rohita at 26% [12]. The supplementation of 10% RSC in pig feeds enhanced growth performance and cost-effectiveness [13]. Additionally, including 20% rubber seed kernel in the concentrate feed of goats did not negatively influence feed intake, nutrient digestibility, rumen fermentation, or nitrogen utilization [14]. The dietary supplementation of 10%-20% yeast-fermented rubber seed kernels for tropical lactating dairy cows [15] and 25% for dairy heifers [16] did not affect feed utilization and microbial protein synthesis. Currently, there is less research on the application of RSC in the diet of Hu sheep compared to fish and dairy cow.

RSC is a nutritionally rich feed resource, especially regarding crude protein and energy. However, effectively integrating RSC into the diet of Hu sheep may reduce the dependence on traditional feedstuffs such as corn and SBM. Therefore, this study aimed to assess the effects of increasing levels of RSC on growth performance, nutrient digestion metabolism, serum biochemical parameters, and rumen microbiota of Hu sheep, to determine the optimal level of RSC supplementation in the diet of Hu sheep. This study provides valuable insights for the application of RSC as a substitute for corn and SBM in the Hu sheep industry.

## Materials and methods

### Experimental materials

The RSC used in the experiment was provided by Yunnan Xishuangbanna Huakun Biotechnology Co. The nutrient contents of the RSC are listed in Table 1. The test animals, 48 Hu sheep, were sourced from Linquan County Xinqun Husbandry Co. Ltd., located in Anhui Province, China.

**Table 1:** Nutrient composition of rubber seed cake (% of DM)

Items	Rubber seed cake
DM	93.67
CP	20.16
EE	6.6
NDF	41.33
ADF	27.24
Ash	2.92
Ca	0.24
P	0.35

*DM* Dry matter, *CP* Crude protein, *NDF* Neutral detergent fiber, *ADF* Acid detergent fiber, *EE* Ether extract

### Animals, diets, and experimental design

48 Hu sheep, evenly split between males and females, weighing 17.01 ± 0.57 kg at 3 months of age, were randomly divided into four treatments: 0% rubber seed cake (RSC0%), 6% rubber seed cake (RSC6%), 12% rubber seed cake (RSC12%) and 18% rubber seed cake (RSC18%). Each group consisted of 12 sheep, with 6 males housed in one pen and 6 females in another. The TMR diets were designed following the Chinese meat sheep feeding standard (NY/T 816–2004). The TMR diets were composed of a 45% forage (30% corn straw silage + 15% peanut vine) to 55% concentrate ratio (DM basis), with clean water available at all times. The composition and nutritional levels of the diets are listed in Table 2. The diets were fed twice a day (07:00 and 17:00). The feeding trial lasted 90 days and consisted of 10 days of adaptation.

**Table 2:** Diet composition and nutritional levels of the four treatments (% of DM)

Items	Diet^a^			
	RSC0%	RSC6%	RSC12%	RSC18%
Ingredient, % of DM				
Corn straw silage	30.00	30.00	30.00	30.00
Peanut vine	15.00	15.00	15.00	15.00
Corn	26.50	21.50	16.50	11.50
Soybean meal	16.00	14.00	12.00	10.00
Rubber seed cake	0.00	6.00	12.00	18.00
Wheat bran	10.00	11.00	12.00	13.00
CaHPO_4_	0.30	0.30	0.30	0.30
NaHCO_3_	0.60	0.60	0.60	0.60
NaCl	0.80	0.80	0.80	0.80
Premix^b^	0.80	0.80	0.80	0.80
Total	100.00	100.00	100.00	100.00
Nutrient levels				
ME^c^, MJ/kg	9.28	9.22	9.15	9.09
DM, %	56.40	56.72	57.36	57.88
CP, %	13.72	13.74	13.82	13.88
NDF, %	34.57	35.28	37.20	37.98
ADF, %	16.53	18.88	20.09	20.47
Ash, %	7.62	7.21	7.11	7.65
EE, %	4.96	4.99	4.98	5.06
Ca, %	0.57	0.59	0.60	0.61
P, %	0.36	0.36	0.37	0.40

*ME* Metabolizable energy, *DM* Dry matter, *CP* Crude protein, *NDF* Neutral detergent fiber, *ADF* Acid detergent fiber, *EE* Ether extract, *Ca* Calcium, *P* Phosphorus

^a^RSC0%: 0% RSC + 26.5% Corn + 16% SBM; RSC6%: 6% RSC + 21.5% Corn + 14% SBM; RSC12%: 12% RSC + 16.5% Corn + 12% SBM; RSC18%: 18% RSC + 11.5% Corn + 10% SBM

^b^The premix provided the following per kg of diets: Vitamin A: 200000 IU/kg, Vitamin D3: 80000 IU/kg, Vitamin E: 200 IU/kg, Fe: 600 mg/kg, Zn: 600 mg/kg, Cu: 100 mg/kg, Mn: 400 mg/kg, I: 40 mg/kg, Co: 25 mg/kg

^c^Metabolizable energy was based on calculated values (NRC, 2011)

### Feed intake and growth performance

Feed intake was meticulously monitored daily for each sheep, with records maintained for both the quantity of feed consumed and any remaining feed (refusals). To ensure accurate measurements, individual body weights were recorded before the morning feeding session at 20-day intervals throughout the study. These data were utilized to calculate the growth performance indicators for each sheep, including the ADG, the DMI, and the F/G (DMI/ADG), which are critical indicators of nutritional efficiency and animal growth. 500 g of feed was collected weekly, dried at 65°C, ground, and then sieved through a 40-mesh sieve for storage and subsequent analysis.

### Determination of nutrient digestion metabolism

On day 72, 6 sheep were randomly selected from each group for digestion and metabolism measurements using the total feces and urine collection method. The sheep were reared individually in a single pen and an acclimatization period of three days preceded a four-day sampling period. During the sampling period, daily fecal and urine samples were weighed, and two portions of 10% of the total amount were taken as representative samples. One aliquot was preserved with the addition of 10 mL of 10% sulfuric acid per 100 g of sample for nitrogen fixation to determine CP, while the other aliquot was reserved for routine nutritional analysis and stored at -20°C.

The DM, CP, EE, Ash, Ca, and P contents of the feed and fecal samples were determined using methods specified by the National Standards of the People's Republic of China. Specifically, GB/T 6435–2014 was used for DM, GB/T 6432–2018 for CP, GB/T 6433–2006 for EE, GB/T 6438–2007 for Ash, GB/T 6436–2018 for Ca, and GB/T 6437–2018 for P. The ADF and NDF concentrations were quantified according to Van Soest et al. [17].

### Serum biochemical parameters

On day 80, 8 sheep from each group were selected and approximately 10 mL of blood was collected by jugular vein sampling, centrifuged at 3,000 g for 20 min at 4°C, and serum collected and stored at -20°C for testing. The following parameters were determined by Nanjing Aoqing Biotechnology Co: GLU (DX3202), UREA (SD2206), TCHO (ZS3172), TG (ZS3170), AST (ZS3010), ALT (ZS3008), TP (DB7122) and ALB (DB7126). The following parameters were measured by ELISA: IgA (H108), IgM (H109), IgG (H106), IL-1β (H002), IL-4 (H005), IL-6 (H007), T-AOC (YH1246), SOD (YH1200), GSH-Px (YH1267) and MDA (YH1217) according to the kit (Nanjing Aoqing Biotechnology Co., Ltd., Jiangsu, China).

### Rumen fermentation characteristics

On day 80, 6 sheep were randomly selected from each group and rumen fluid was collected by oral fasting and filtered through four layers of gauze. The pH was immediately determined using a portable pH meter (S220-K). The rumen fluid was cryopreserved at -80°C for subsequent analysis. Ruminal NH_3_-N was determined using a phenol-hypochlorite method [1]. The VFA was measured utilizing the gas chromatograph (A91Plus, Changzhou Pano Instrument Co., Ltd., China) as described by Ran et al. [18].

### Rumen microbiota analysis

A total of 24 frozen rumen fluid samples (6 samples in each group) were sent to Sangon BioTech (shanghai) for 16S rDNA sequencing analysis, as described in the online Supplementary method S1.

### Statistical analysis

Data analysis was performed using one-way ANOVA to evaluate the effect of four diets with varying levels of RSC on the designated parameters, followed by post-hoc comparisons using Duncan's multiple range test, as implemented in SPSS version 20.0 software. Polynomial contrasts were applied to evaluate the linear and quadratic trends associated with varying levels of dietary RSC supplementation. Results are presented as the mean and SEM. Statistical significance was set at a threshold of *P* < 0.05.

## Results

### Growth performance

As presented in Table 3, there were no treatment effects on total body weight gain, ADG, DMI, and F/G among the experimental groups (*P* > 0.05). However, when compared to the RSC0%, the ADG increased by 2.21% in the RSC6% and decreased by 2.91% in the RSC18%.

**Table 3:** Effects of rubber seed cake on the growth performance of Hu sheep

Items	Diet^a^				SEM	*P*-value		
	RSC0%	RSC6%	RSC12%	RSC18%		ANOVA	Linear	Quadratic
BW, kg								
d 1	16.70	17.28	16.90	17.17	0.57	0.984	0.838	0.966
d 80	28.01	28.84	28.40	28.15	0.51	0.944	0.976	0.861
Total body weight gain, kg	11.31	11.56	11.50	10.98	0.25	0.878	0.687	0.708
ADG, g/d	141.41	144.53	143.75	137.29	3.15	0.878	0.687	0.708
DMI, g/d	905.47	916.31	908.00	918.92	42.64	0.996	0.936	0.997
F/G	6.56	6.40	6.38	6.98	0.28	0.889	0.638	0.731

*BW* body weight, *ADG* average daily gain, *DMI* dry matter intake, *F/G* DMI/ADG

^a^RSC0%: 0% RSC + 26.5% Corn + 16% SBM; RSC6%: 6% RSC + 21.5% Corn + 14% SBM; RSC12%: 12% RSC + 16.5% Corn + 12% SBM; RSC18%: 18% RSC + 11.5% Corn + 10% SBM

### Nutrient apparent digestibility

As presented in Table 4, supplementing RSC had no significant effect on the apparent digestibility of DM, CP, NDF, or ADF in Hu sheep (*P* > 0.05). The apparent digestibility of OM and EE quadratically (*P* < 0.05) changed with the increase of RSC supplementation, with the greatest apparent digestibility of OM and EE observed in the RSC6% diet.

**Table 4:** Effects of rubber seed cake on the nutrient apparent digestion of Hu sheep

Items, %	Diet^1^				SEM	*P*-value		
	RSC0%	RSC6%	RSC12%	RSC18%		ANOVA	Linear	Quadratic
DM	70.47	70.86	68.53	68.21	0.49	0.100	0.028	0.085
OM	73.48^a^	73.73^a^	70.72^b^	70.41^b^	0.47	0.018	< 0.001	< 0.001
CP	69.90	70.41	69.82	68.53	0.41	0.418	0.201	0.241
EE	86.60^ab^	87.06^a^	86.24^ab^	84.16^b^	0.43	0.049	0.018	0.019
NDF	51.56	50.82	48.69	48.37	0.77	0.383	0.091	0.239
ADF	49.66	48.88	46.28	46.17	1.31	0.726	0.271	0.541

*DM* Dry matter, *OM* Organic matter, *CP* Crude protein, *EE* Ether extract, *NDF* Neutral detergent fiber, *ADF* Acid detergent fiber

^a,b^Mean values with different small letter superscripts mean significant difference (*P* < 0.05)

^1^RSC0%: 0% RSC + 26.5% Corn + 16% SBM; RSC6%: 6% RSC + 21.5% Corn + 14% SBM; RSC12%: 12% RSC + 16.5% Corn + 12% SBM; RSC18%: 18% RSC + 11.5% Corn + 10% SBM

### Nitrogen metabolism

As presented in Table 5, the N intake and fecal N increased linearly (*P* < 0.05), and the apparent digestibility of N decreased linearly with increased RSC supplementation (*P* < 0.001).

**Table 5:** Effects of rubber seed cake on the nitrogen metabolism of Hu sheep

Items	Diet^1^				SEM	*P*-value		
	RSC0%	RSC6%	RSC12%	RSC18%		ANOVA	Linear	Quadratic
N intake, g/d	26.90^c^	27.62^bc^	28.49^ab^	28.86^a^	0.18	< 0.001	< 0.001	< 0.001
Fecal N, g/d	7.55^b^	8.15^ab^	8.86^a^	9.04^a^	0.15	0.001	< 0.001	< 0.001
Urinary N, g/d	6.85	5.99	6.79	6.95	0.15	0.101	0.431	0.185
Deposition N, g/d	12.50	13.49	12.85	12.87	0.18	0.256	0.762	0.376
Utilization rate of N, %	46.80	48.97	45.31	44.73	0.77	0.212	0.154	0.244
Apparent digestibility of N, %	72.10^a^	70.54^ab^	69.04^b^	68.71^b^	0.39	0.005	< 0.001	0.002

^a,b,c^Mean values with different small letter superscripts mean significant difference (*P* < 0.05)

^1^RSC0%: 0% RSC + 26.5% Corn + 16% SBM; RSC6%: 6% RSC + 21.5% Corn + 14% SBM; RSC12%: 12% RSC + 16.5% Corn + 12% SBM; RSC18%: 18% RSC + 11.5% Corn + 10% SBM

### Serum biochemical, immunological, and antioxidant parameters

**Fig. 1:**
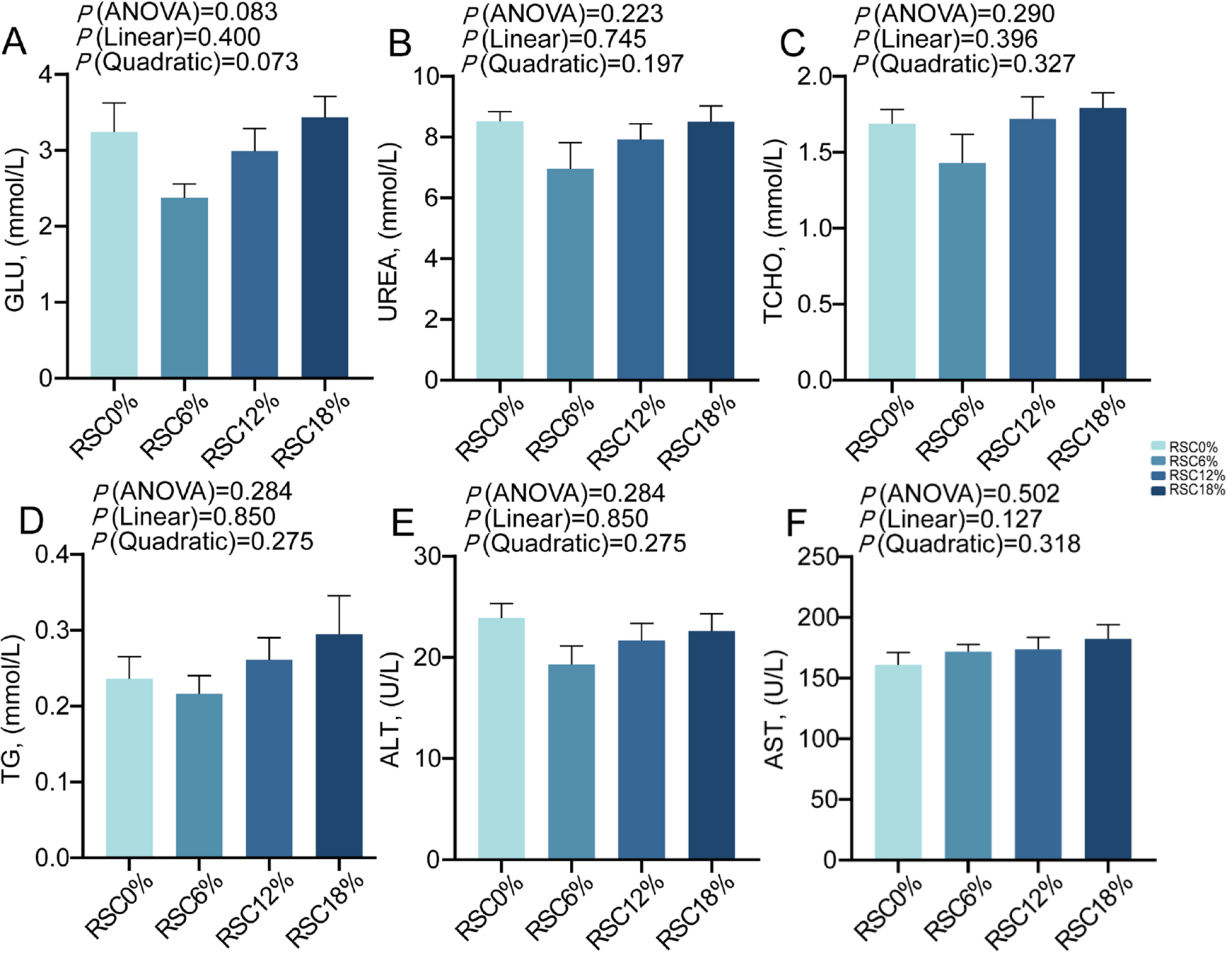
Effects of rubber seed cake on the serum biochemical parameters of Hu sheep. Values are presented as mean ± SEM, *n* = 8 per group. Serum GLU level (**A**), serum UREA level (**B**), serum TCHO level (**C**), serum TG level (**D**), serum ALT level (**E**), serum AST level (**F**)

**Fig. 2:**
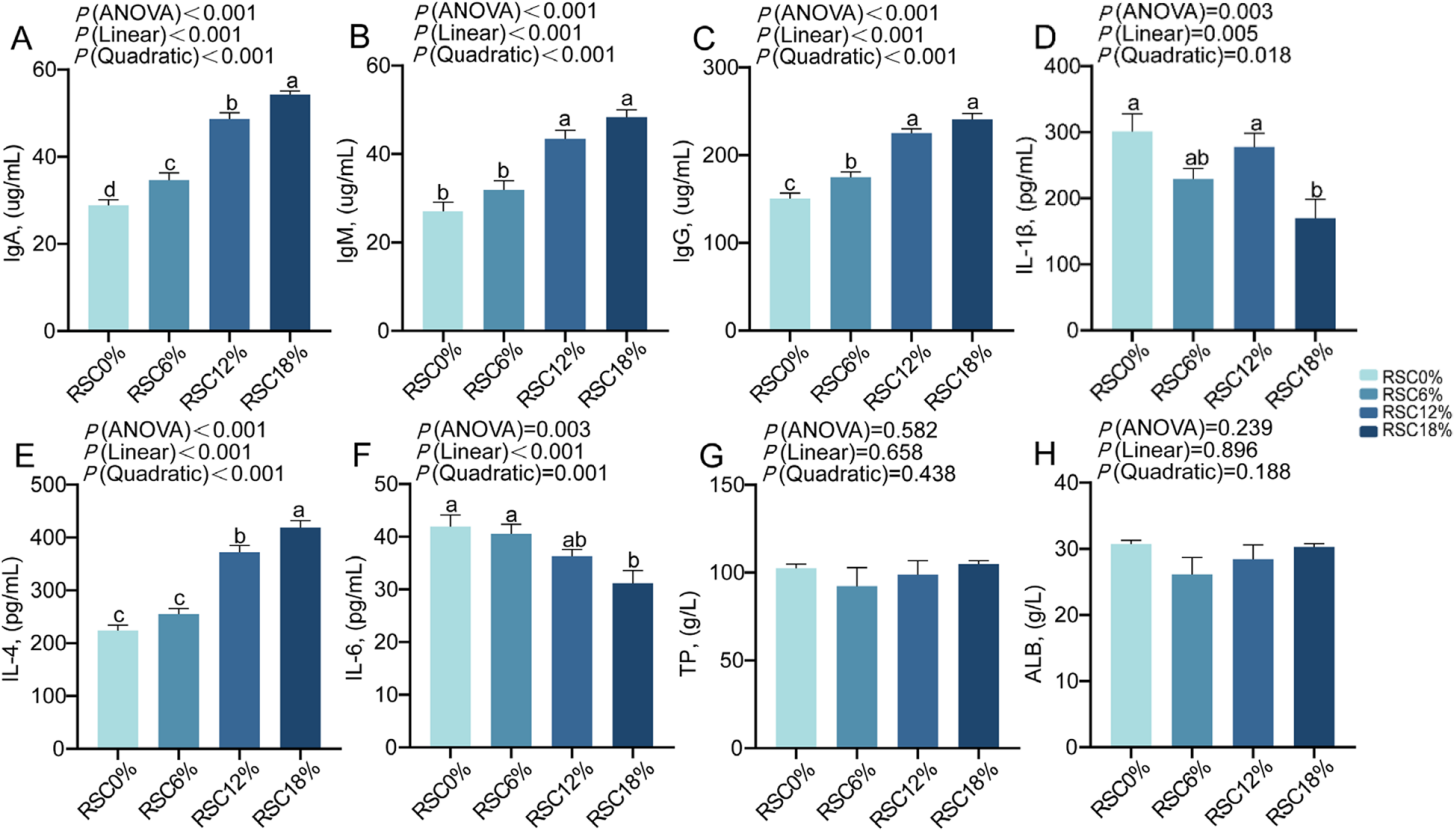
Effects of rubber seed cake on the serum immunological parameters of Hu sheep. Values are presented as mean ± SEM, n = 8 per group. ^a−d^ Mean values with different small letter superscripts mean significant difference (*P* < 0.05). Serum IgA level (**A**), serum IgM level (**B**), serum IgG level (**C**), serum IL-1β level (**D**), serum IL-4 level (**E**), serum IL-6 level (**F**), serum TP level (**G**), serum ALB level (**H**)

**Fig. 3:**
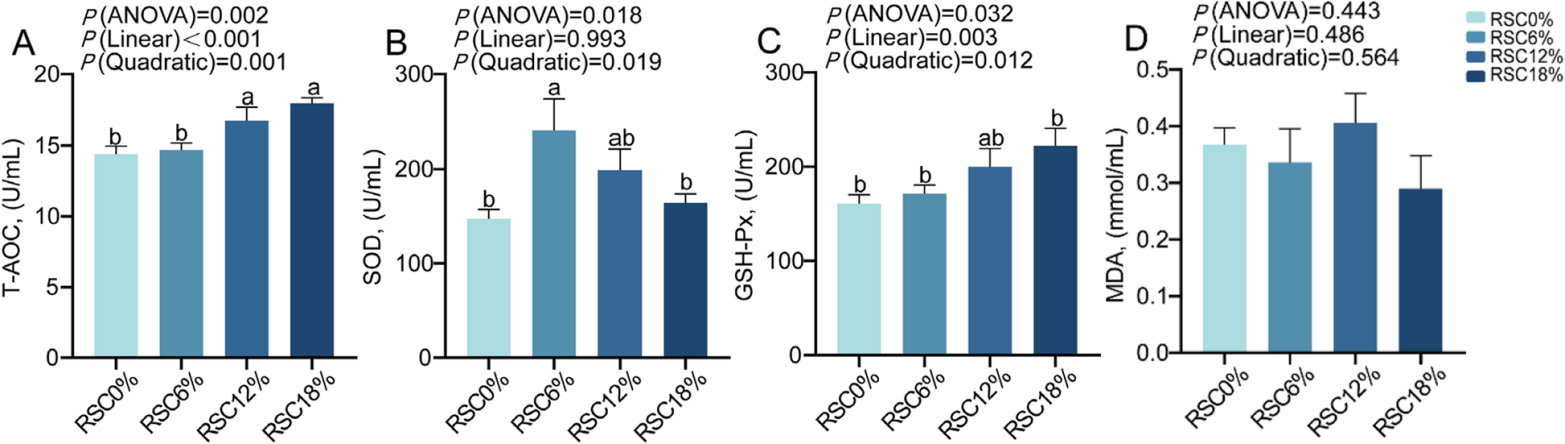
Effects of rubber seed cake on the serum antioxidant parameters of Hu sheep. Values are presented as mean ± SEM, n = 8 per group. ^a−b^ Mean values with different small letter superscripts mean significant difference (*P* < 0.05). Serum T-AOC level (**A**), serum SOD level (**B**), serum GSH-Px level (**C**), serum MDA level (**D**)

As presented in Fig. 1, there were no treatment effects on the serum levels of GLU, UREA, TCHO, TG, ALT, and AST (*P* > 0.05). As presented in Fig. 2, the serum levels of IgA, IgM, IgG, and IL-4 increased linearly with increased RSC supplementation (*P* < 0.05). With increased RSC supplementation, there was a linear reduction in the serum level of IL-6 (*P* < 0.001), and the serum level of IL-1β reduced quadratically (*P* = 0.018). As presented in Fig. 3, with increased RSC supplementation, the serum levels of GSH-Px and T-AOC increased linearly (*P* < 0.05); The serum level of SOD increased quadratically (*P* = 0.019) with the increased RSC dose.

### Rumen fermentation characteristics

As presented in Table 6, the supplementation of RSC had no significant effect on rumen fluid pH, acetate, propionate, butyrate, and A/P (*P* > 0.05). NH_3_-N quadratically (*P* < 0.001) changed with the increase of RSC supplementation.

**Table 6:** Effects of rubber seed cake on rumen fermentation characteristics of Hu sheep

Items	Diet^1^				SEM	*P*-value		
	RSC0%	RSC6%	RSC12%	RSC18%		ANOVA	Linear	Quadratic
pH	7.22	7.10	7.16	7.19	0.02	0.292	0.112	0.270
NH_3_-N, mg/100mL	16.28^ab^	14.72^b^	14.00^b^	17.73^a^	0.50	0.030	0.868	< 0.001
Acetate, mmol/L	33.61	33.70	34.75	34.83	1.18	0.978	0.672	0.917
Propionate, mmol/L	8.57	8.14	7.72	7.69	0.29	0.717	0.254	0.502
Butyrate, mmol/L	6.03	5.53	5.31	5.76	0.30	0.789	0.649	0.587
A/P	3.95	4.16	4.52	4.55	0.12	0.265	0.050	0.144

^a,^^b^Mean values with different small letter superscripts mean significant difference (*P* < 0.05)

^1^RSC0%: 0% RSC + 26.5% Corn + 16% SBM; RSC6%: 6% RSC + 21.5% Corn + 14% SBM; RSC12%: 12% RSC + 16.5% Corn + 12% SBM; RSC18%: 18% RSC + 11.5% Corn + 10% SBM

### Rumen microbiota

**Fig. 4:**
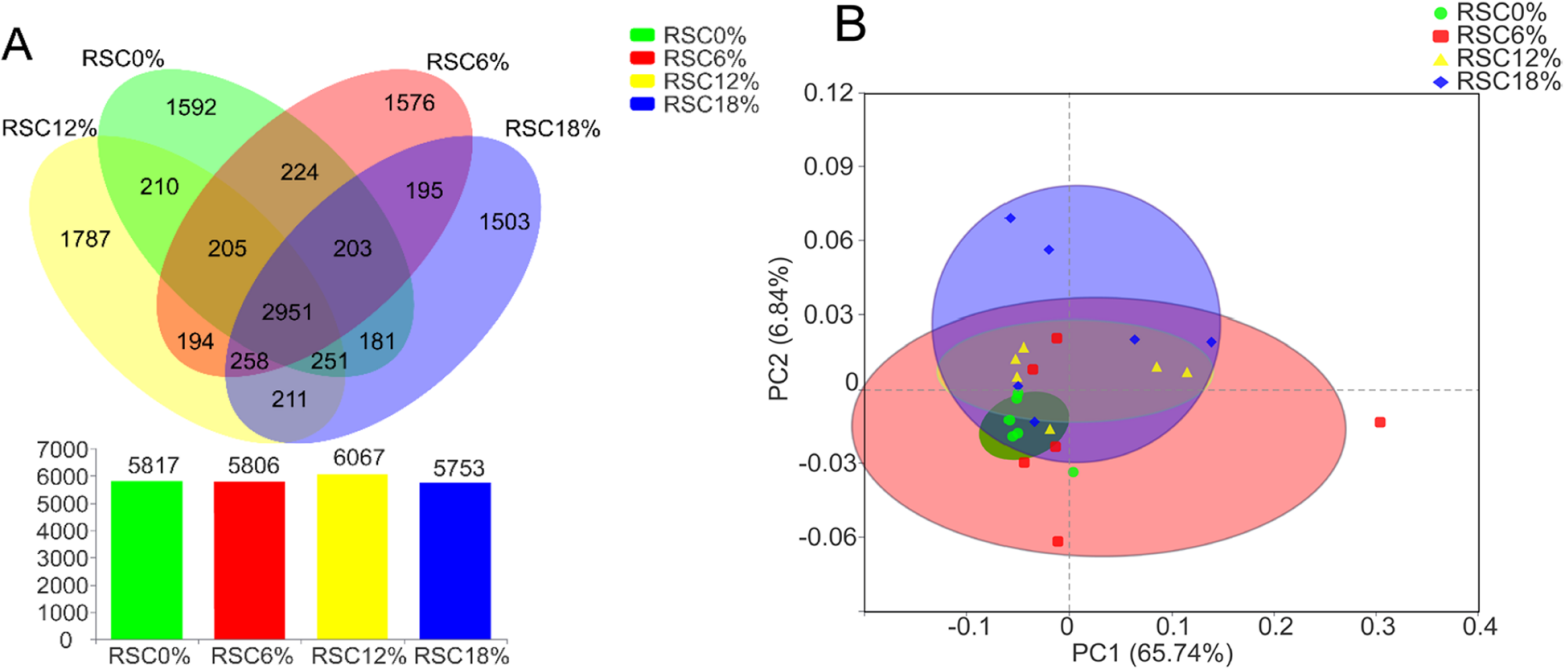
Effects of rubber seed cake on rumen microbiota of Hu sheep. **A** Venn diagram showing the unique and shared OTUs in the different groups; **B** Unweighted Unifrac PCoA analysis based on OTUs of rumen microbiota

A total of 499,918, 524,447, 513,507, and 518,805 effective sequences were identified in rumen samples from the RSC0%, RSC6%, RSC12%, and RSC18% groups, respectively. A Venn analysis of OTUs revealed that 5,817, 5,806, 6,067, and 5,753 OUTs were detected in the RSC0%, RSC6%, RSC12%, and RSC18% groups, respectively. Additionally, 2,951 OTUs were shared among the four groups. A total of 1592, 1576, 1787, and 1503 OTUs were identified as unique to the RSC0%, RSC6%, RSC12%, and RSC18% groups, respectively (Fig. 4A). To examine the beta diversity of the bacterial communities, an unweighted Unifrac PCoA was conducted to illustrate the differentiation of community composition among the treatments (Fig. 4B). The alpha diversity of the rumen microbiota is shown in Table 7, the dietary treatments exerted no significant influence on the ACE, Chao1, Shannon, and Simpson indexes.

**Table 7:** The alpha diversity in the rumen microbiota of Hu sheep

Items	Diet^a^				SEM	*P*-value		
	RSC0%	RSC6%	RSC12%	RSC18%		ANOVA	Linear	Quadratic
ACE	3,111.45	3,102.87	3,133.72	3,117.84	37.59	0.994	0.886	0.989
Chao 1	2,870.51	2,851.62	2,901.15	2,913.50	33.45	0.923	0.562	0.829
Shannon	6.02	5.58	5.79	5.78	0.08	0.304	0.481	0.336
Simpson	0.01	0.03	0.02	0.02	0.01	0.498	0.741	0.566

^a^RSC0%: 0% RSC + 26.5% Corn + 16% SBM; RSC6%: 6% RSC + 21.5% Corn + 14% SBM; RSC12%: 12% RSC + 16.5% Corn + 12% SBM; RSC18%: 18% RSC + 11.5% Corn + 10% SBM

The relative abundances at the phylum level (relative abundance > 1%) are presented in Supplementary Fig. S1A, the top 5 dominated phyla were *Bacteroidota*, *Firmicutes*, *Proteobacteria*, *Euryarchaeota,* and *Planctomycetota*. At the genus level, the top 20 genera are shown in Supplementary Fig. S1B.

As presented in Supplementary Table S1, At the genus level (TOP20), *Rikenellaceae_RC9_gut_group*, *Christensenellaceae_R-7_group*, *norank_Muribaculaceae*, and *norank_F082* were the main dominant genera. The relative abundance of *norank_Muribaculaceae* quadratically (*P* = 0.012) changed with increased RSC supplementation, with the greatest relative abundance of *norank_Muribaculaceae* observed in the RSC6% diet.

**Fig. 5:**
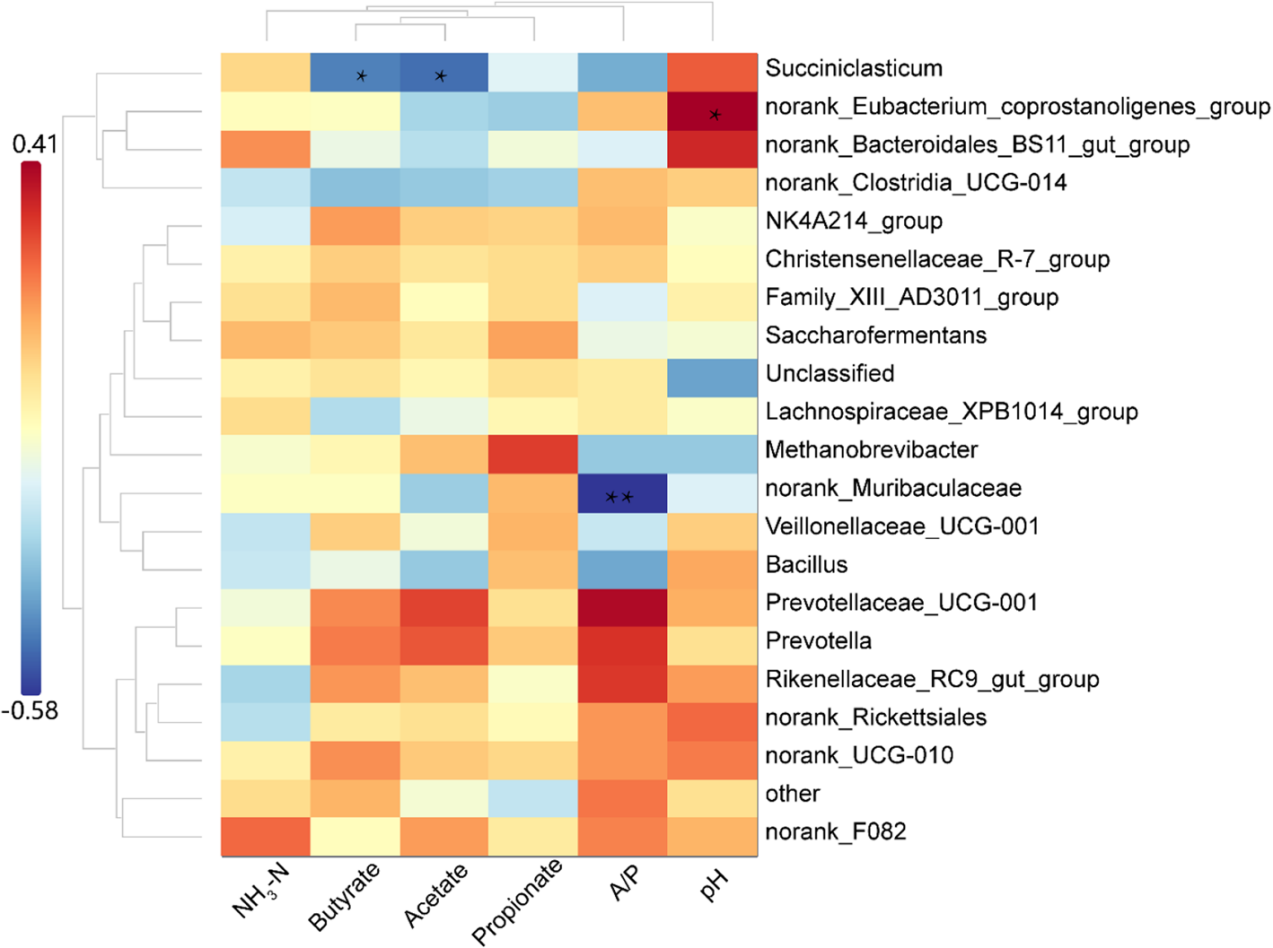
Correlation between rumen microbiota (at genus level) and rumen fermentation characteristics. Positive correlations (red circles) and negative correlations (blue circles) were illustrated in the heatmap by Spearmen correlation analysis. (* means *P* < 0.05; ** means *P* < 0.01)

A Spearman correlation analysis was conducted to explore the relationship between the relative abundance of the top 20 rumen genera and ruminal fermentation characteristics (Fig. 5). A significant negative correlation between *Succiniclasticum* and the concentrations of acetate and butyrate (*P* < 0.05). Additionally, a significant positive correlation was observed between the *norank_Eubacterium_coprostanoligenes_group* and rumen pH (*P* < 0.05). Furthermore, *norank_Muribaculaceae* showed a significant negative correlation with the A/P (*P* < 0.01).

## Discussion

The supplementation of different levels of RSC in the diet of Hu sheep had no effect on DMI, indicating that RSC has no impact on the palatability of the diet. Chanjula et al. [14] demonstrated that goats can utilize up to 20% of rubber seed kernel without exhibiting any adverse effects on feed intake. Increasing the level of yeast-fermented rubber seed kernel in the diet of dairy cows did not affect feed intake [15]. Similarly, the inclusion of defatted or fermented rubber seed meal at appropriate levels in the feed did not affect the feed intake of Nile tilapia [11], common carp [19], and hybrid tilapia [10]. Furthermore, ADG demonstrated an increase of 2.21% in the RSC6%, while exhibiting a decrease of 2.91% in the RSC18% when compared to the RSC0%. The results demonstrate that the incorporation of a moderate quantity of RSC proved advantageous for the growth outcomes of Hu sheep, consistent with supplementing 15% RSC to the diet will not harm the growth performance of *Hemibagrus wyckioides*, while higher substitution levels can lead to negative impacts [9]. The negative impact of a high level of RSC on the growth of Hu sheep may be attributed to the presence of phytic acid in RSC [12]. The chelating nature of phytic acid reduces the digestibility and retention rate of feed nutrients in animals, thereby inhibiting the growth rate of the organism [20]. The reason for the improved growth performance of Hu sheep with the incorporation of 6% RSC is partly attributed to the enhancement of DMI, and on the other hand, it may be associated with improved nutrient digestion metabolism. Consequently, we experimented to investigate the effect of RSC on the apparent digestibility and nitrogen metabolism of Hu sheep.

The present study found that the incorporation of 6% level RSC into the diet enhanced the apparent digestibility of OM and EE in Hu sheep. Gunun et al. [16] demonstrated that the supplementation of yeast-fermented rubber seed in dairy cattle diets had no effect on the apparent digestibility of DM, CP, NDF, or ADF, but improved the apparent digestibility of EE. In addition, Chanjula et al. [14] indicated that the supplementation of rubber seed kernel to goat diets had no effect on the apparent digestibility of DM, OM, and CP, but also improved the apparent digestibility of EE. Nitrogen utilization may reflect the utilization of dietary protein and amino acids by animal digestion, absorption, and utilization. In this study, the N intake and fecal N increased, but the apparent digestibility of N reduced with the increased levels of RSC supplementation. Previous research results indicated that feeding goats with rubber seed kernel reduced total nitrogen intake and fecal nitrogen while improving nitrogen retention [14]. The higher CP, EE apparent digestibility, N deposition, and N utilization of Hu sheep in the RSC6% than in the other groups explained the highest ADG in the RSC6%. As the level of supplementation increased, apparent nutrient digestibility decreased in the RSC12% and RSC18%, which may be related to the fact that RSC contains a small number of anti-nutritional factors, including cyanogenic glycoside [21] and tannin [9].

The supplementation of different levels of RSC in the diet of Hu sheep improved their immune and antioxidant capabilities. Immunoglobulins (IgA, IgG, and IgM) play a crucial role in the body's humoral immune response, safeguarding against pathogenic invasion, preserving bodily homeostasis, and serving as indicators of immune system functionality [22]. In the present study, supplementing different levels of RSC to the diet can increase the levels of IgA, IgM, and IgG in the serum of Hu sheep. The increase in immunoglobulins in the serum may be related to the rich content of amino acids and trace minerals in RSC, especially the zinc content, which is three times that of SBM. Studies have found that Zn-Gly can improve the levels of immunoglobulins in the serum of broilers [23]. IL-1β, a pro-inflammatory cytokine exerting localized effects on immune cells, is capable of potentiating immune responses and mitigating stress-induced cellular damage. However, excessive secretion of IL-1β can lead to tissue damage and consequently impair immune system functionality [24]. IL-4 is a typical immunomodulatory cytokine that is of considerable importance in immune regulation, allergic responses, and cancer therapy [25]. IL-6 is a crucial pro-inflammatory cytokine that is involved in the body's inflammatory responses and plays a significant role in immune regulation, the nervous system, and hematopoiesis [26, 27]. Supplementing different levels of RSC to the diet increased the serum level of IL-4 and reduced the serum levels of IL-1β and IL-6. T-AOC reflects the overall level of various antioxidants and antioxidant enzymes in the body [28]. SOD and GSH-Px are two crucial antioxidant enzymes within the organism, responsible for modulating the production and elimination of free radicals, thereby preventing cellular damage caused by lipid peroxides [29]. In this study, the supplementation of different levels of RSC in the diet increased the serum levels of GSH-Px, T-AOC, and SOD. Previous study results indicate that the supplementation of 15%-30% RSC in the diet of *Hemibagrus wyckioides* had no effect on the levels of SOD and GSH-Px in the liver, and reduced the levels of MDA in the plasma [9]. The improvement in immune and antioxidant levels in Hu sheep fed with RSC may be attributed to the rich content of trace minerals in RSC. Trace minerals are believed to enhance the immune and antioxidant capabilities of Tibetan sheep [30], but the exact mechanisms require further investigation.

Rumen pH is a pivotal indicator of rumen environment stability and the status of rumen fermentation, primarily influenced by VFA, NH_3_-N, and salivary secretions [31]. The normal range for rumen pH is generally accepted to be between 6.0 and 7.5 [32]. The rumen fluid pH values of Hu sheep in all four treatment groups were within the normal range (7.10 to 7.22). NH_3_-N reflects the equilibrium between the rate of nitrogen degradation by the rumen microbiota and nitrogen utilization, as well as the extent to which rumen microbes utilize nutrients from the ration [33]. The appropriate concentration of NH_3_-N is 5–30 mg/100 mL [34]. In this study, the NH_3_-N ranged from 14.00 to 17.73 mg/100 mL, which can support microbial growth. Volatile fatty acids produced by rumen fermentation are more than 70% of the body's energy requirement and are also the main carbohydrate source for rumen microorganisms [35]. Acetic acid is the main substance for synthesizing body fat and milk fat. Propionic acid serves as a key gluconeogenic precursor in animals, predominantly originating from the microbial degradation of non-structural carbohydrates within the feed [36]. Butyric acid, after being converted to β-hydroxybutyric acid in the rumen, is metabolized in the liver and thus provides energy for the growth and development of the organism [37, 38]. This study found that supplementing different levels of RSC to the diet did not affect VFA in the rumen, which is consistent with previous research that reported yeast-fermented rubber seed kernel could be used as a replacement for components such as soybean meal in the diets of dairy heifers without negatively affecting the production of VFA in the rumen [16]. The above results indicate that RSC can be incorporated into the diet of Hu sheep without adversely affecting rumen fermentation.

The rumen microbiome is crucial for the digestive system of ruminant animals and comprises three major groups: bacteria, protozoa, and fungi [39]. The presence of these microbial communities enables ruminants to effectively digest cellulose and synthesize a large amount of microbial protein, which becomes the main source of nutrition for ruminants [40]. Modifying the dietary composition can significantly enhance ruminal microbial diversity and optimize rumen fermentation in ruminants, thereby augmenting animal productivity and economic profitability [41]. In the rumen of ruminant animals, *Firmicutes* and *Bacteroidota* account for more than 70% of the rumen microorganisms [42–44]. In this study, the combined proportion of *Bacteroidota* and *Firmicutes* colonies across the four groups constituted over 80% of the rumen microbial community. *Bacteroidota* mainly degrade structural polysaccharides and proteins [45], *Bacteroidota* has a broad metabolic potential and can quickly adapt to the available nutrients in the gastrointestinal microbiota, producing succinate, acetate, and butyrate, as the main end-products [40, 46]. *Firmicutes* are a major group of fiber-decomposing bacteria that can convert fibrous materials in feed into volatile fatty acids, providing energy for the life activities of ruminant animals [47, 48]. *Proteobacteria* can degrade soluble carbohydrates, but high levels (> 19%) indicate an unstable rumen ecosystem [49]. In this study, *Proteobacteria* levels were under 19%, indicating a stable ecosystem. Furthermore, supplementing RSC in the diet of Hu sheep can enhance the relative abundance of *Bacteroidota*, which explains that feeding RSC improves the immunity ability of Hu sheep. This provides some evidence that RSC can improve the ruminal microbial composition in Hu sheep.

At the genus level, *Rikenellaceae_RC9_gut_group*, *Christensenellaceae_R-7_group*, *F082*, and *Muribaculaceae* were the dominant rumen genera similar to the results of Wang et al. [50]. *Christensenellaceae_R-7_group* can secrete three types of glycosidases that facilitate the breakdown of cellulose and hemicellulose in forage feed, thus enhancing feed utilization efficiency [51]. *Christensenellaceae_R-7_group* is linked to the health and digestive processes of ruminants, playing a significant role in butyric acid production [52] and the enhancement of rumen development [53]. However, *Christensenellaceae* was found to be inversely associated with body weight [54]. We hypothesize that a decrease in the relative abundance of the *Christensenellaceae_R-7_group* may correlate with an increase in body weight for Hu sheep. In this study, *norank_Muribaculaceae* was the highest in the RSC6% and was significantly negatively correlated with A/P. Metagenomic analyses predict that between 75–100% of species within the *Muribaculaceae* family are capable of synthesizing aspartate, glutamine, glutamate, and other amino acids [55]. *Muribaculaceae* are capable of metabolizing both endogenous and exogenous polysaccharides, encompassing α-glucans, plant-derived sugars, and host sugars [56], and *Muribaculaceae* have an anti-inflammatory effect [57]. Furthermore, *Succiniclasticum* showed a significant negative correlation with acetate and butyrate levels. This indicates a close correlation between rumen microbiota and VFA production.

## Conclusions

In summary, these results indicated that incorporating RSC into the diet of Hu sheep did not adversely affect growth performance and rumen fermentation characteristics. Supplementing with 6% RSC enhanced the relative abundance of *norank_Muribaculacea* in the rumen fluid and the immune and antioxidant capabilities of the sheep. However, supplementing with 12 and 18% RSC might have negatively impacted nutrient digestion and metabolism. Therefore, this study recommended replacing corn and soybean meal with 6% RSC in the diet of Hu sheep.

## Supplementary Information

Supplementary Material 1: Supplementary method S1. 16S rDNA sequencing analysis. Supplementary Fig. S1. Effects of rubber seed cake on phylum and genus of rumen microbiome of Hu sheep. Relative abundances at phylum (A) and genus(B). Supplementary Table S1. Effects of rubber seed cake on the relative abundance at the phylum (relative abundance > 1%) and genus (TOP20) of rumen microbiome of Hu sheep.

## Data Availability

The metagenome sequences obtained from Hu lamb rumen fluids have been deposited in the NCBI Short Read Archive (GenBank accession number: PRJNA1140428).

## Abbreviations

SBM: Soybean meal
RSC: Rubber seed cake
ADG: Average daily gain
DMI: Dry matter intake
OM: Organic matter
N: Nitrogen
ME: Metabolizable energy
DM: Dry matter
CP: Crude protein
NDF: Neutral detergent fiber
ADF: Acid detergent fiber
EE: Ether extract
GLU: Glucose
TCHO: Total cholesterol
TG: Triglyceride
AST: Aspartate aminotransferase
ALT: Alanine aminotransferase
TP: Total protein
ALB: Albumin
IgA: Immunoglobulin A
IgM: Immunoglobulin M
IgG: Immunoglobulin G
IL-1β: Interleukin-1β
IL-4: Interleukin-4
IL-6: Interleukin-6
T-AOC: Total antioxidant capacity
SOD: Superoxide dismutase
GSH-Px: Glutathione peroxidase
MDA: Malondialdehyd
