# Supplementary material for: Effects of increasing levels of rubber seed cake on growth performance, nutrient digestion metabolism, serum biochemical parameters, and rumen microbiota of Hu sheep
Source: BMC Vet Res. 2025 Feb 5;21:52. doi: 10.1186/s12917-025-04503-7 (PMC11796160; doi:10.1186/s12917-025-04503-7)
Supplement: Supplementary file 1 — Supplementary Material 1: Supplementary method S1. 16S rDNA sequencing analysis. Supplementary Fig. S1. Effects of rubber seed cake on phylum and genus of rumen microbiome of Hu sheep. Relative abundances at phylum (A) and genus(B). Supplementary Table S1. Effects of rubber seed cake on the relative abundance at the phylum (relative abundance > 1%) and genus (TOP20) of rumen microbiome of Hu sheep. [file 12917_2025_4503_MOESM1_ESM.docx]

**Effects of increasing levels of rubber seed cake on growth performance, nutrient digestion metabolism, serum biochemical parameters, and rumen microbiota of Hu sheep**

**Supplementary material**

1. 16S rDNA sequencing analysis

The samples underwent DNA extraction, and the polymerase chain reaction (PCR) was initiated immediately after the DNA was extracted. The 16S rRNA V3–V4 amplicon was amplified using 2×Hieff Robust PCR Master Mix (Yeasen, 10105ES03, China). Two universal bacterial 16S rRNA gene amplicon PCR primers (PAGE purified) were used: the amplicon PCR forward primer (CCTACGGGNGGCWGCAG) and amplicon PCR reverse primer (GACTACHVGGGTATCTAAT-CC). The PCR products were analyzed by agarose gel electrophoresis (2%) and used Hieff NGSTM DNA Selection Beads (Yeasen, 10105ES03, China) to purify the free primers and primer dimer species in the amplicon product. Sequencing was performed using the Illumina MiSeq system (Illumina MiSeq, USA). After sequencing, the two short Illumina readings were assembled by PEAR software (version 0.9.8) according to the overlap, and fastq files were processed to generate individual fasta and qual files, which could then be analyzed by standard methods. The effective tags were clustered into operational taxonomic units (OTUs) of ≥ 97% similarity using Usearch software (version 11.0.667).

Venn diagrams, PCoA analysis, and Spearman’s correlation analysis were completed using the online data visualization and analysis tool(https://ngs.sangon.com).

1. Effects of rubber seed cake on phylum and genus of rumen microbiome of Hu sheep. Relative abundances at phylum (A) and genus(B).


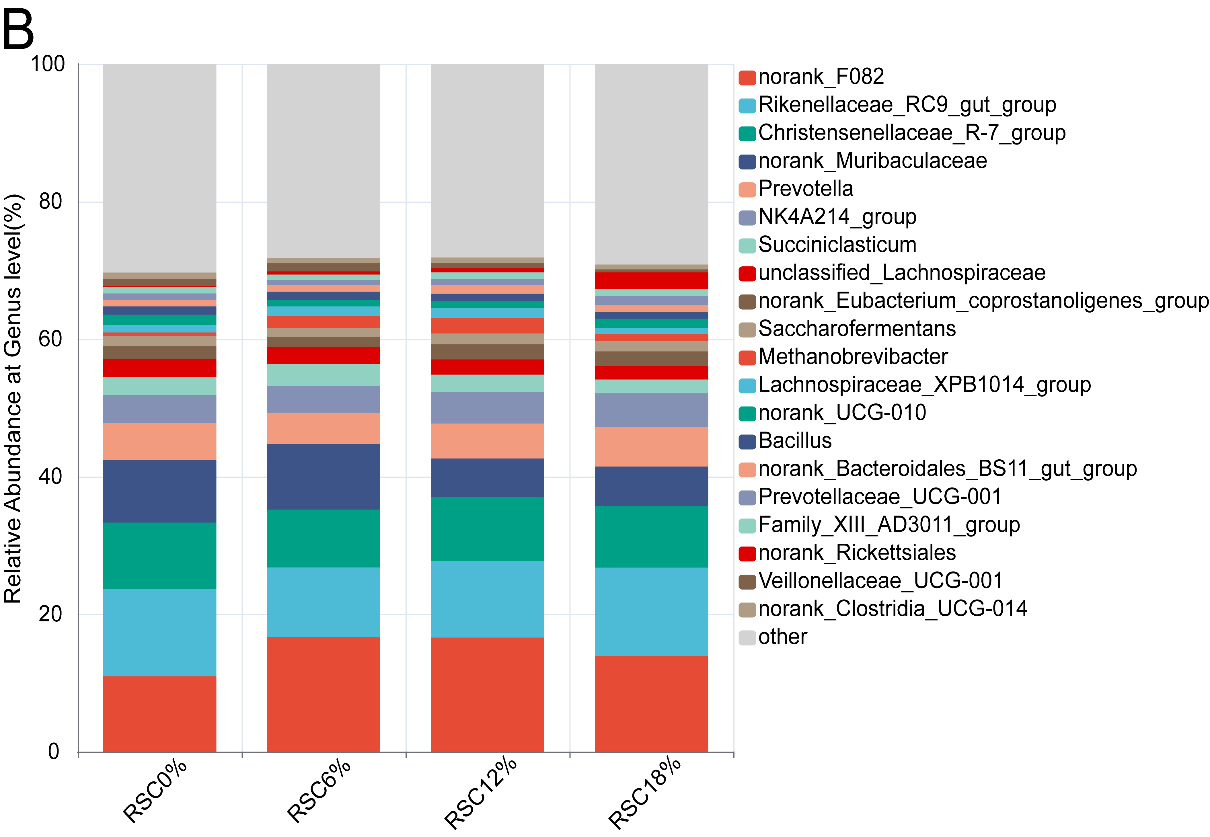

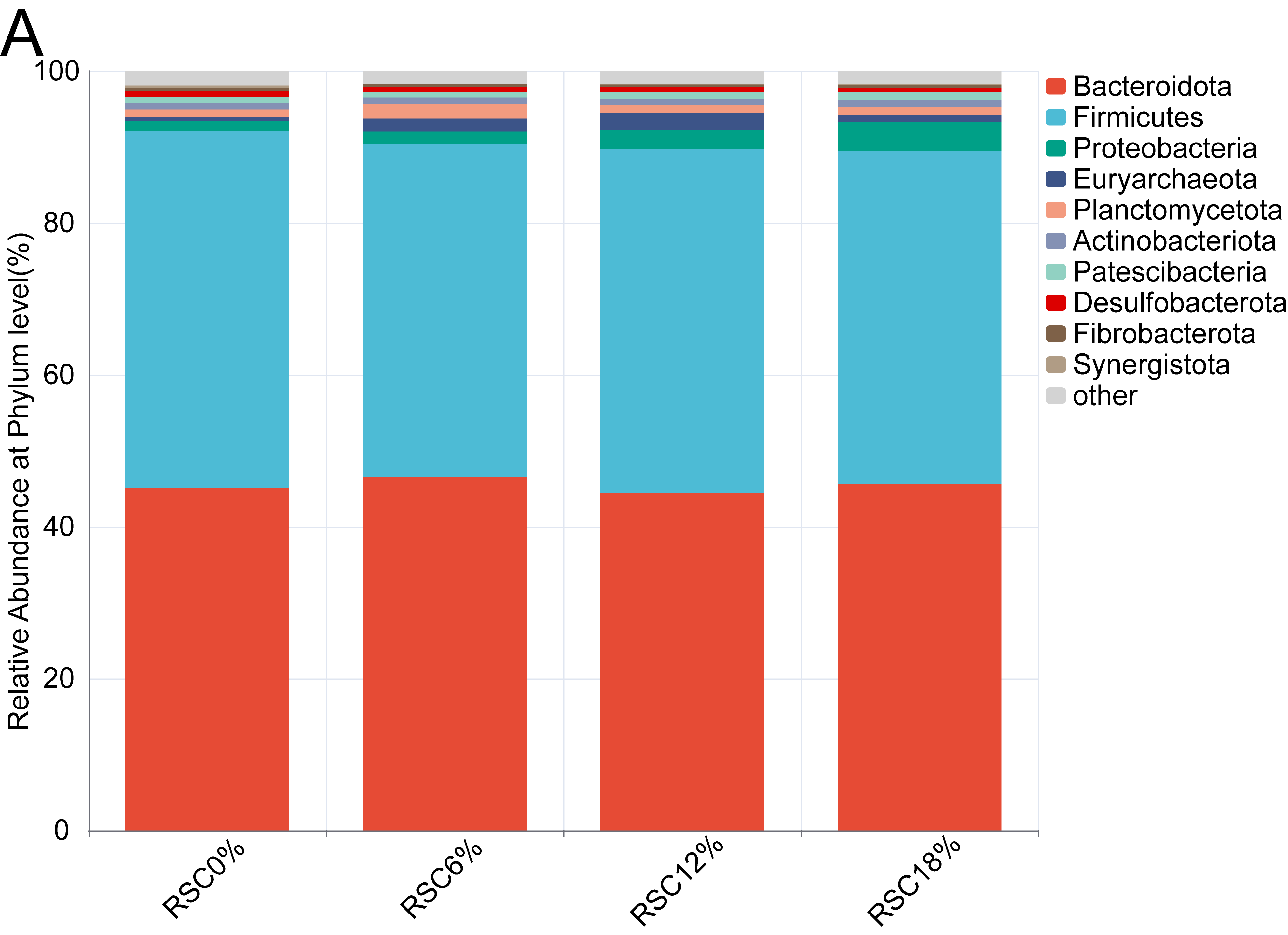


1. Effects of rubber seed cake on the relative abundance at the phylum (relative abundance >1%) and genus (TOP20) of rumen microbiome of Hu sheep.

| Items | Diet^1^ | | | | SEM | *P*-value | | |
| --- | --- | --- | --- | --- | --- | --- | --- | --- |
|  | RSC0% | RSC6% | RSC12% | RSC18% |  | ANOVA | Linear | Quadratic |
| Phylum |  |  |  |  |  |  |  |  |
| *Firmicutes* | 46.93 | 43.83 | 45.23 | 43.80 | 1.11 | 0.747 | 0.433 | 0.693 |
| *Bacteroidota* | 45.10 | 46.52 | 44.46 | 45.63 | 1.32 | 0.961 | 0.970 | 0.998 |
| *Proteobacteria* | 1.40 | 1.68 | 2.51 | 3.80 | 0.46 | 0.263 | 0.050 | 0.130 |
| *Planctomycetota* | 1.04 | 1.91 | 0.97 | 1.03 | 0.20 | 0.293 | 0.595 | 0.531 |
| *Euryarchaeota* | 0.46 | 1.72 | 2.30 | 1.01 | 0.31 | 0.154 | 0.430 | 0.076 |
| Genus |  |  |  |  |  |  |  |  |
| *norank_F082* | 10.99 | 16.66 | 16.61 | 13.91 | 1.63 | 0.588 | 0.560 | 0.381 |
| *Rikenellaceae_RC9_gut_group* | 12.69 | 10.15 | 11.11 | 12.87 | 0.61 | 0.351 | 0.795 | 0.212 |
| *Christensenellaceae_R-7_group* | 9.64 | 8.41 | 9.34 | 8.92 | 0.49 | 0.847 | 0.791 | 0.893 |
| *norank_Muribaculaceae* | 9.13^b^ | 9.56^b^ | 5.59^a^ | 5.77^a^ | 0.56 | 0.005 | 0.003 | 0.012 |
| *Prevotella* | 5.37 | 4.55 | 5.07 | 5.78 | 0.41 | 0.785 | 0.648 | 0.605 |
| *NK4A214_group* | 4.09 | 3.91 | 4.64 | 4.87 | 0.26 | 0.560 | 0.200 | 0.416 |
| *Succiniclasticum* | 2.54 | 3.11 | 2.48 | 2.02 | 0.34 | 0.757 | 0.489 | 0.602 |
| *unclassified_Lachnospiraceae* | 2.64 | 2.43 | 2.19 | 1.97 | 0.13 | 0.323 | 0.056 | 0.168 |
| *norank_Eubacterium_coprostanoligenes_ group* | 1.94 | 1.57 | 2.21 | 2.11 | 0.11 | 0.200 | 0.261 | 0.455 |
| *Saccharofermentans* | 1.48 | 1.28 | 1.58 | 1.48 | 0.11 | 0.840 | 0.767 | 0.939 |
| *Methanobrevibacter* | 0.46 | 1.72 | 2.30 | 1.00 | 0.31 | 0.154 | 0.429 | 0.075 |
| *Lachnospiraceae_XPB1014_group* | 1.10 | 1.42 | 1.41 | 0.96 | 0.08 | 0.109 | 0.546 | 0.046 |
| *norank_UCG-010* | 1.49 | 0.94 | 0.98 | 1.32 | 0.10 | 0.134 | 0.594 | 0.061 |
| *Bacillus* | 1.22 | 1.19 | 1.12 | 0.98 | 0.24 | 0.320 | 0.223 | 0.166 |
| *norank_Bacteroidales_BS11_gut_group* | 0..98 | 0.96 | 1.27 | 0.98 | 0.15 | 0.872 | 0.818 | 0.885 |
| *Prevotellaceae_UCG-001* | 0.90 | 0.74 | 0.88 | 1.36 | 0.14 | 0.468 | 0.245 | 0.272 |
| *Family_XIII_AD3011_group* | 0.92 | 0.79 | 0.93 | 1.05 | 0.40 | 0.763 | 0.477 | 0.599 |
| *norank_Rickettsiales* | 0.18 | 0.46 | 0.60 | 2.37 | 0.40 | 0.207 | 0.060 | 0.111 |
| *Veillonellaceae_UCG-001* | 1.01 | 1.19 | 0.74 | 0.44 | 0.16 | 0.376 | 0.128 | 0.238 |
| *norank_Clostridia_UCG-014* | 0.94 | 0.76 | 0.82 | 0.71 | 0.28 | 0.566 | 0.238 | 0.484 |

^a–b^ Mean values with different small letter superscripts mean significant difference (*P* < 0.05). ^1^ RSC0%: 0% RSC + 26.5% Corn + 16% SBM; RSC6%: 6% RSC + 21.5% Corn + 14% SBM; RSC12%: 12% RSC + 16.5% Corn + 12% SBM; RSC18%: 18% RSC + 11.5% Corn + 10% SBM.
